# The anti-adhesive and anti-aggregatory effects of phenolics from *Trifolium* species in vitro

**DOI:** 10.1007/s11010-015-2620-y

**Published:** 2015-12-19

**Authors:** Joanna Kolodziejczyk-Czepas, Malgorzata Sieradzka, Barbara Wachowicz, Pawel Nowak, Wieslaw Oleszek, Anna Stochmal

**Affiliations:** Department of General Biochemistry, Faculty of Biology and Environmental Protection, University of Lodz, Pomorska 141/143, 90-236 Lodz, Poland; Department of Biochemistry, Institute of Soil Science and Plant Cultivation, State Research Institute, Czartoryskich 8, 24-100 Pulawy, Poland

**Keywords:** Adhesion, Aggregation, Clover, Platelets, *Trifolium*

## Abstract

The present in vitro study includes a comparative evaluation of anti-platelet (anti-thrombotic) properties of plant phenolics, isolated from nine different clover (*Trifolium*) species. The analysis covered phenolic fractions isolated from *T. alexandrinum* L., *T. fragiferum* L., *T. hybridum* L., *T. incarnatum* L., *T. pallidum* Waldst et Kit., *T. resupinatum* L. var. *majus* Boiss, *T. resupinatum* L. var. *resupinatum*, *T. scabrum* L., and *T. pratense* L. (red clover). The inhibitory effects of plant preparations (1–50 µg/ml) on hemostatic functions of blood platelets were assessed by measurements of thrombin- or ADP-induced platelet adhesion to fibrinogen, platelet aggregation in platelet-rich plasma (activated with ADP or collagen), and by the determination of PF-4 secretion from platelet *α*-granules. The influence of *T.* phenolics on arachidonic cascade in blood platelets was also determined. *T. resupinatum* var. *majus*, *T. resupinatum* var. *resupinatum,* and *T. scabrum* had the strongest anti-platelet effects. These preparations displayed the most evident anti-adhesive and anti-aggregatory effects in response to all of the used agonists: thrombin (0.2 U/ml), ADP (10 µM), and collagen (2 µg/ml), and their inhibitory properties were also confirmed by an analysis of PF-4 secretion. *T. scabrum* and some of other examined clover species possess significantly higher concentrations of both isoflavones and other bioactive phenolics, when compared to red clover. The obtained results suggest that these clovers contain substances with potent anti-platelet properties.

## Introduction

Folk medicine recommendations have been a starting point for research on dietary supplements and new remedies for clinical use. Ethnomedicinal application of up to 80 % of plant-derived drugs may be the same or similar to the current use of the active substances of those species [1]. Clovers are mostly identified as valuable forage plants; however, both *T. pratense* (the best known of *Trifolium* species) and other species from the *Trifolium* genus are characterized by a wide range of therapeutic uses in folk medicine of different world regions. Some clovers are also listed as traditional wild food [2, 3]. Moreover, the latest findings suggest that edible flower extracts of *T. pratense* L. and *T. repens* L. may be promising functional components of human diet [4]. The curative properties of red clover have been evidenced by numerous ethnomedicinal surveys as well as by in vitro and in vivo examinations [5]. Nowadays, red clover is a bioactive ingredient of numerous dietary supplements, nutraceuticals, or herbal drugs, commonly used in order to mitigate menopausal complaints. Moreover, it has been found that herbal preparations originated from this plant display the cardiovascular disease-preventive effects [6]. So far, *T. pratense* has been mainly studied in terms of isoflavone content and phytoestrogenic properties. Simultaneously, it is well known that *Trifolium* species are a rich source of other (poly)phenols that may determine biological activities of preparations from these plants. Our earlier comparative analysis of 57 *Trifolium* species [7] demonstrated the presence of three or four main groups of polyphenolic substances such as flavonoids, phenolic acids, and clovamides, in clover extracts.

Under physiological conditions, platelet activation and accumulation at sites of vascular injury are crucial stage of haemostasis, preventing the blood loss. On the other hand, it is also well established that the enhanced platelet activation (e.g., associated with numerous western diseases) may lead to the formation of pathogenic thrombi. Therefore, the research on natural compounds with anti-platelet and/or anticoagulation properties have been intensively developed [8]. Numerous epidemiological studies indicated the importance of diet rich in plant-derived polyphenols in the prevention of cardiovascular diseases. The disease-preventive effects of natural substances are mainly attributed to their antioxidant properties, protecting against the harmful effects of oxidative stress, which is frequently associated with occurrence of cardiovascular risk factors [9–11]. Another aspect of beneficial influence of plant substances on human health may be their ability to prevent the hyperactivation of blood platelets. The enhanced platelet activation and oxidative stress are important factors, involved in the pathogenesis of numerous cardiovascular disorders and complications of other diseases. Thus, in the prevention of Western diseases, the combination of antioxidant and anti-platelet activity of plant extract is particularly desirable from the pharmacological point of view [12–14]. For instance, both radical scavenging and anti-platelet effects were found for an ethnomedicinal plant *Salvia miltiorrhiza* Bunge *S.miltiorrhiza* has been used extensively in traditional medicine in China and Korea in the treatment of coronary heart disease, cerebrovascular disease, and inflammation. Scientific studies confirmed its anti-thrombotic properties, including the inhibition of ADP and collagen-induced platelet aggregation as well as thrombin-stimulated platelet adhesion to collagen or fibrinogen [15]. The inhibitory effects on platelet aggregation were also found for other plants, including *Allium sativum* [16], *Cyperus rotundus* [17], *Ficus glomerata* [18], and *Urtica dioica* [19].

The present work is based on the in vitro evaluation of anti-platelet (anti-thrombotic and cardioprotective) properties of plant preparations from nine clover *Trifolium* species, i.e., phenolic fractions of *T. alexandrinum* Jusl*, T. fragiferum* L.*, T. hybridum* L.*, T. incarnatum* L.*, T. pallidum* Waldst et Kit.*, T. pratense* L., *T. resupinatum* L. var. *majus* Boiss*, T. resupinatum* L. var. *resupinatum,* and *T. scabrum* L. The plant extracts were examined as possible sources of cardioprotective substances for future use in dietary supplements or herbal drugs. In previous studies, we demonstrated considerable free radical scavenging properties and antioxidant effects of the above species [20–23]. The biological activity of clovers other than *T. pratense* and their influence on human health have been not well described yet. The existing evidence indicates the antioxidant, anti-inflammatory, anti-diabetic, and anti-cancer properties of some clovers [5]; however, the comparative evaluation of the anti-platelet (anti-thrombotic) actions of extracts isolated from several different species is a novel aspect of physiological effects of *Trifolium*-derived substances. Until recently, very few studies have been designed to define the effects of *Trifolium* plants on blood components, including the haemostatic system. Red clover isoflavones were found to activate synthesis of an anti-platelet factor: nitric oxide (NO), by stimulation of transcriptional pathways in endothelial cells [24]. Furthermore, Lam et al. [25] reported that isoflavones obtained from red clover might suppress inflammation.

## Materials and methods

### Chemicals

For measurements of platelet adhesion, the Thermo Scientific Pierce BCA Protein Assay kit (Thermo Scientific, Rockford, USA) was used. ADP and collagen were from Chrono-Log Corporation (Havertown, USA), and thrombin was purchased from BioMed Lublin, Poland. Fibrinogen was isolated from human plasma, according to the method described by Doolittle et al. [26]. All other organic and nonorganic reagents (of an analytical grade) were obtained from local commercial suppliers.

### Plant material

Seeds of clover: *T*. *alexandrinum* Jusl, *T*. *fragiferum* L., *T*. *hybridum* L., *T*. *incarnatum* L., *T*. *pallidum* et Kit., *T*. *pretense* L., *T*. *resupinatum* L. var. *majus* Boiss, *T*. *resupinatum* L. var. *resupinatum,* and *T*. *scabrum* L. derived from the genebank, Zentralinstitute für Pflanzengenetik und Kulturpflanzenforschung (Gatersleben, Germany) were sown on experimental plots of the Institute of Soil Science and Plant Cultivation in Pulawy, Poland. The herbarium voucher numbers are as follows, respectively: TRIF 30/79, TRIF 37/83, TRIF 6/82, TRIF 82/83, TRIF 253/95, TRIF 186/75, TRIF 81/83, TRIF 43/80, and TRIF 120/79. Above-ground parts of plants were collected at the beginning of flowering, frozen, freeze-dried, ground, and used in the preparation of extracts.

### Extraction and fractionation of extracts

Each of the nine *Trifolium* species was extracted according to the method previously described, consisting in maceration of the plant material with an aqueous solution of methanol (80 % (v/v) MeOH) at room temperature for 24 h [27]. After low-pressure evaporation, extracts were fractionated in accordance with method of Stochmal et al. [27]. In short, the extract was dissolved in water and was applied to a short preparative column packed with reversed-phase C18 package. First, in order to remove sugars, the column was washed with water and then using 40 % MeOH, phenolic fraction was eluted, which contained phenolic acids, flavonoids, isoflavones, and clovamides. The quantitative content of these compounds was determined by UPLC with photodiode array detector (PDA) [7], and the results are shown in Table 1.

**Table 1:** The phytochemical characterization of the examined phenolic fractions isolated from aerial parts of nine *Trifolium* species [22, 23]

*Trifolium* species	Concentration (mg/g of dry mass)				
	Total phenolic content	Clovamides	Flavonoids	Isoflavones	Phenolic acids
*T. alexandrinum*	52.55	9.63	22.30	18.97	1.65
*T. hybridum*	15.24	0.44	5.22	–	9.58
*T. fragiferum*	11.30	–	5.18	5.50	0.62
*T. incarnatum*	47.97	–	41.54	5.10	1.32
*T. pallidum*	35.06	12.94	0.61	7.31	14.20
*T. resupinatum* var. *resupinatum*	17.32	–	11.25	5.21	0.86
*T. resupinatum* var. *majus*	22.54	–	9.76	11.17	1.61
*T. scabrum*	80.93	–	7.67	72.76	0.66
*T. pratense*	14.82	–	3.89	5.01	5.92

### Isolation of blood platelets and preparation of the examined samples

Blood from healthy volunteers was purchased from the Regional Centre of Blood Donation and Blood Treatment in Lodz, Poland. All experiments were approved by the committee on the Ethics of Research at the University of Lodz (KBBN-UL/II/18/2012).

Platelet-rich plasma (PRP) and blood platelets were isolated by differential centrifugation of blood [28]. The sedimented platelets were then suspended in the Tyrode’s buffer; the amount platelets were estimated spectrophotometrically, accordingly to the method of Walkowiak et al. [29]. For adhesion measurements, 1.8–2.2 × 10^8^ platelets/ml were used, while the determination of malondialdehyde (MDA) was performed with the use of 5 × 10^8^ of platelets/ml. The stock solutions of *Trifolium* (10–20 mg/ml) phenolic fractions were prepared in 20 or 30 % DMSO. The preliminary measurements of the activity of native platelets and platelets treated with DMSO at concentrations corresponding to those added with *Trifolium* phenolic fractions revealed that DMSO itself had no effect. However, in order to maintain the same experimental conditions, DMSO was added to all control samples. Thus, activities of platelets pre-incubated with *Trifolium* fractions were compared to the DMSO-containing controls. Samples were pre-incubated with the examined *Trifolium* preparations (at the final concentrations of 1–50 µg/ml), for 15 min at 37 °C, and subsequently used for experiments.

### Platelet adhesion

Adhesion of blood platelets to fibrinogen (0.1 mg/ml) was determined by a static method, according to Tuszynski and Murphy [30]. A 96-well, flat-bottom microplates were coated with 100 µl of adhesive protein suspension (fibrinogen) for overnight, at 4 °C. Then, the nonadsorbed protein was removed by three-time washing with 250 µl of 0.1 M TBS buffer. Then, 200 µl of 1 % BSA (a heat-shock fraction) in TBS was added to each well. The microtiter plates were incubated for 2 h at 37 °C. The BSA remnants were then removed by three-time washing with 250 µl of TBS. The platelet suspensions (100 µl of DMSO-containing control or samples pre-incubated with the *Trifolium* preparations) were added into the wells. Immediately, 50 µl of the agonist was added to the wells (to obtain the final concentration of 10 µM for ADP and 0.2 U/ml for thrombin, respectively). After an hour of incubation at 37 °C, the non-adherent platelets were removed by three-time washing with TBS, and then, 200 µl of the BCA mixture was added. After 60 min incubation at 37 °C, the absorbance at 560 nm was measured.

### Platelet aggregation

Platelet aggregation was measured in PRP, using the Chrono-Log 490 aggregometer. PRP samples were pre-incubated of 15 min at 37 °C with the examined *Trifolium* preparations and transferred into aggregometer cuvettes. Platelet aggregation was induced by ADP (at the final conc of 10 μM) or collagen (at the final conc of 2 μg/ml).

### Platelet factor 4 secretion

The procedure was performed using of the RayBiotech© Human PF-4 ELISA kit. The secretion of PF-4 was monitored in PRP, after stimulation by ADP (10 µM, final conc) or collagen (0.2 µg/ml, final conc). PRP samples (control and pre-incubated with the examined phenolic fractions) were activated by the agonists (10 min, 37 °C), and then, immediately centrifuged in order to remove blood platelets. Before the PF-4 immunodetection, platelet poor plasma (PPP) was diluted 200 times with phosphate buffered saline.

### Arachidonic acid metabolism—measurements of MDA generation

To 0.5 ml of platelet suspensions (5 × 10^8^ ml), in Tyrodes’s buffer, thrombin was added to the final conc of 0.4 U/ml. After 5 min of incubation, equal volumes of trichloroacetic acid (15 %, in 0.25 M HCl) and thiobarbituric acid (0.37 %, in 0.25 M HCl) were added. After 2 min of vigorous shaking, the samples were boiled for 10 min, cooled, and centrifuged in order to sediment the platelet pellet. A clear supernatant was collected. Colorimetric measurements were performed at *λ* = 535 nm. The concentration of MDA was calculated from the molar absorption coefficient for the thiobarbituric acid-reactive substances (*ε* = 156 000 M^−1^ × cm^−1^) [31].

### Statistical analysis

The first step of statistical analysis was the elimination of the uncertain data by the *Q*-Dixon test. All the values in this work are expressed as mean ± SEM; *p* values <0.05 are considered to be statistically significant; *n* = number of blood donors (at least two independent pre-incubations of clover extracts with blood platelets or PRP from each donor were performed). The used tests are indicated in figure legend and table captures.

## Results

In the first stage of the study, effects of the examined *Trifolium* preparations (1, 5, and 50 µg/ml) on platelet adhesion (stimulated by thrombin or ADP) were assessed (Table 2). Measurements of thrombin-induced adhesion of isolated platelets revealed the most evident inhibitory effect of phenolic fractions of *T. incarnatum*, *T. resupinatum* var. *majus,* and *T. scabrum*. During evaluations of platelet response to 10 µM ADP, the phenolics of *T. incarnatum*, *T. resupinatum* var. *majus*, *T. scabrum* as well as *T. pratense* displayed significantly higher anti-adhesive effects.

**Table 2:** The inhibitory effects of phenolic fractions of nine *Trifolium* species on blood platelet adhesion to fibrinogen in vitro

*Trifolium* species	Concentration (µg/ml)	Platelet adhesion to fibrinogen (%)	
		Platelets stimulated with thrombin (0.2 U/ml)	Platelets stimulated with ADP (10 μM)
Control (untreated) platelets	0	100 ± 7.06	100 ± 3.08
*T. alexandrinum*	1	93.00 ± 5.57	87.14 ± 1.98
	5	85.61 ± 5.11	89.73 ± 3.56
	50	85.56 ± 3.77	83.96 ± 2.75^*^
*T. hybridum*	1	86.96 ± 4.25	88.36 ± 2.43
	5	85.81 ± 4.73	88.37 ± 1.81
	50	88.99 ± 4.77	88.55 ± 2.61
*T. fragiferum*	1	90.40 ± 4.26	89.90 ± 3.62
	5	92.16 ± 4.47	90.32 ± 3.56
	50	86.70 ± 4.93	82.49 ± 1.72^**^
*T. incarnatum*	1	83.48 ± 4.06	80.87 ± 2.53^*^
	5	84.12 ± 5.57^*^	76.38 ± 3.99^***^
	50	73.27 ± 4.38^**^	77.82 ± 1.86^***^
*T. pallidum*	1	80.89 ± 4.47^*^	85.16 ± 4.49^*^
	5	76.36 ± 4.28^*^	87.26 ± 3.19
	50	84.19 ± 4.05	87.70 ± 3.36
*T. resupinatum* var. *resupinatum*	1	86.07 ± 5.02	84.48 ± 4.11^*^
	5	90.29 ± 4.78	85.63 ± 3.80^*^
	50	80.30 ± 4.88^*^	80.25 ± 2.37^***^
*T. resupinatum* var. *majus*	1	81.25 ± 4.56	77.42 ± 4.21^***^
	5	75.65 ± 3.54^**^	73.99 ± 2.88^***^
	50	78.46 ± 6.27^**^	75.00 ± 2.88^***^
*T. scabrum*	1	71.28 ± 4.32^***^	83.04 ± 4.99^**^
	5	76.76 ± 4.86^**^	80.43 ± 4.57^**^
	50	83.77 ± 3.89	81.74 ± 3.70^**^
*T. pratense*	1	90.72 ± 3.05	79.93 ± 3.10^***^
	5	84.40 ± 3.43	82.18 ± 3.76^**^
	50	81.21 ± 4.48	80.36 ± 2.90^***^

The anti-adhesive actions of *Trifolium* phenolics were assessed in comparison to the control (untreated) platelets, which adhesion was assumed as 100 %; * *p* < 0.05, ** *p* < 0.01, *** *p* < 0.001, as evaluated by the Dunnett test; *n* = 10–12

**Fig. 1:**
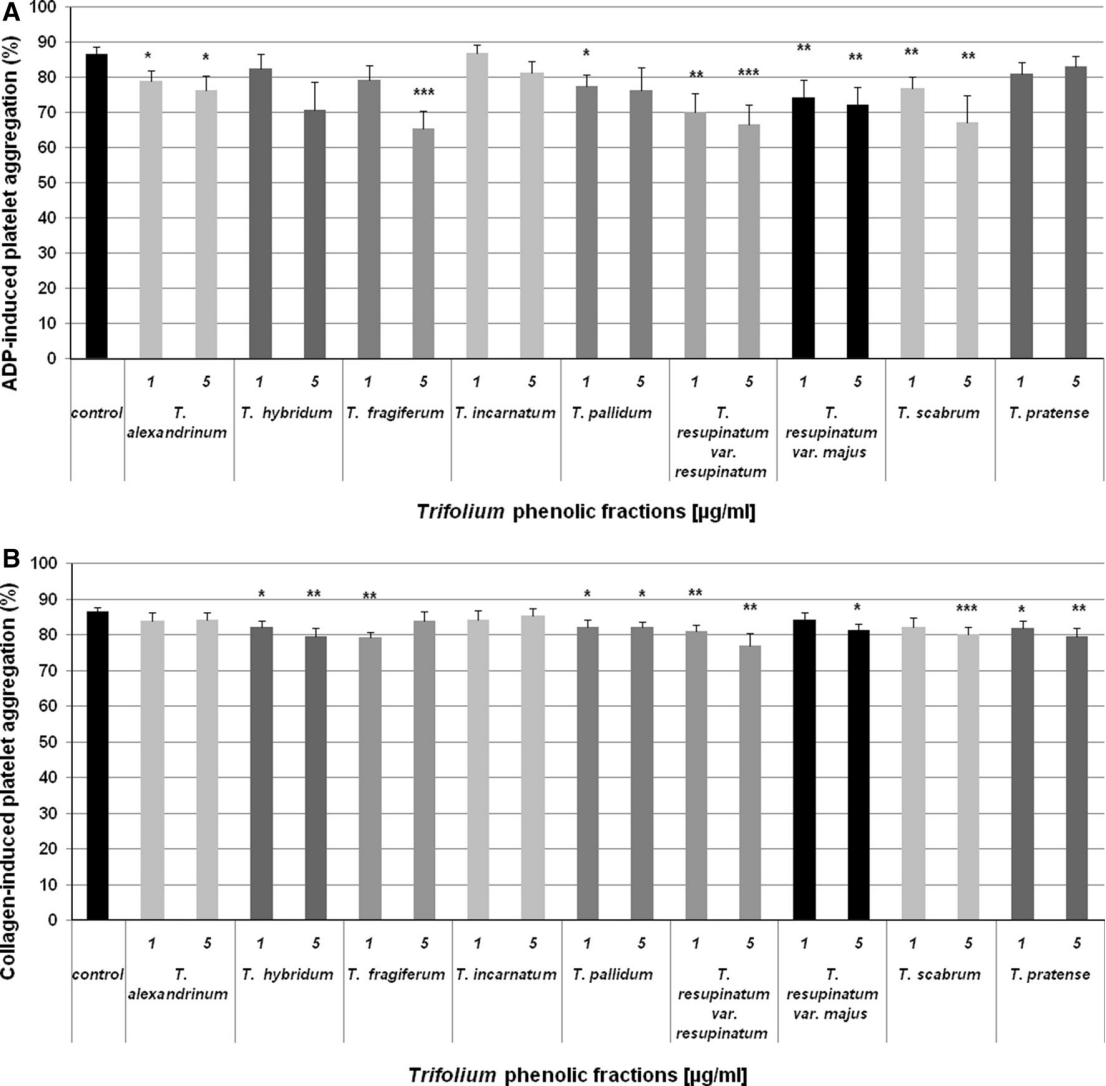
Anti-aggregatory properties of phenolic fractions isolated from nine *Trifolium* species. Platelet aggregation was measured in PRP, after stimulation by ADP (10 µM; panel A) or collagen (2 µg/ml; panel B); * *p* < 0.05, ** *p* < 0.01, *** *p* < 0.001, as evaluated by the *t*-Student’s or Wilcoxon tests; *n* = 8

The next part of this work included determination of abilities of *Trifolium* preparations (1 and 5 µg/ml) to inhibit platelet aggregation (Fig. 1a, b). At this stage, platelet response to agonists (ADP and collagen) was assessed in PRP. After the stimulation with ADP, the most potent inhibitor of platelet aggregation was the phenolic fraction of *T. resupinatum* var. *resupinatum*. The aggregation of blood platelets was also significantly reduced by preparations of *T. resupinatum* var. *majus*, *T. scabrum,* and *T. alexandrinum* (1–5 µg/ml). Anti-platelet actions of *T. fragiferum* and *T. pallidum* were slightly weaker, while for *T. hybridum*, *T. incarnatum,* and *T. pratense,* no statistically significant inhibition was found (Fig. 1a). In experiments with the collagen-induced platelet aggregation, inhibitory actions of the examined *Trifolium* species were comparable, except the preparations of *T. alexandrinum* and *T. incarnatum,* which had no anti-platelet effects in this assay (Fig. 1b).

After comparison of the anti-adhesive and anti-aggregatory effects of the examined *Trifolium* preparations, the additional measurements of collagen or ADP-induced PF-4 secretion from platelet *α* granules were performed with only one, selected concentration of the *Trifolium* preparations (5 µg/ml). These experiments revealed some differences between anti-platelet actions of the examined clover species (Table 3). Determination of the ADP-induced secretion of PF-4 confirmed anti-platelet properties of *T. scabrum*, which were also observed in platelet aggregation measurements. Additionally, this assay showed the inhibitory effect of *T. pratense*, which was not detected during platelet aggregation. The reduction of PF-4 release from platelets in the presence of *T. resupinatum* var. *resupinatum, T. resupinatum* var. *majus,* and *T. pallidum* phenolics was also observed; however, it was statistically insignificant. Furthermore, some inhibitory effects were recorded for samples treated with phenolic fractions of *T. resupinatum* var. *resupinatum*, *T. resupinatum* var. *majus*, *T. scabrum,* and *T. pallidum* in the measurements of the collagen-induced PF-4 release, but the statistical significance of these differences was not found (Table 3).

**Table 3:** Evaluation of inhibitory action of phenolic fractions from nine *Trifolium* species on platelet factor 4 (PF-4) secretion in vitro (*n* = 8 and *n* = 6 for ADP and collagen, respectively

*Trifolium* species [5 μg/ml]	ADP-induced secretion of PF-4 [ng/ml]	Collagen-induced secretion of PF-4 [ng/ml]
−Control platelets	185.522 ± 43.434	360.697 ± 78.792
*T. alexandrinum*	195.885 ± 45.266	331.368 ± 76.012
*T. hybridum*	212.905 ± 45.229	334.229 ± 74.290
*T. fragiferum*	180.445 ± 34.190	346.132 ± 62.437
*T. incarnatum*	156.345 ± 28.480	294.535 ± 33.036
*T. pallidum*	125.513 ± 21.196	315.051 ± 42.761
*T. resupinatum* var. *resupinatum*	124.997 ± 26.417	249.117 ± 51.367
*T. resupinatum* var. *majus*	156.472 ± 26.929	252.156 ± 33.623
*T. scabrum*	132.423 ± 22.464^*^	271.010 ± 35.424
*T. pratense*	117.140 ± 16.849^*^	263.485 ± 33.555

* *p* < 0.05, as evaluated by the Dunnett and *t*-Student’s tests)

Furthermore, the analysis of MDA formation from PGH_2_ in platelets stimulated by thrombin revealed a statistically significant inhibition of platelet secretory process in samples treated with phenolic fractions of *T. alexandrinum*, *T. hybridum*, *T. incarnatum*, *T. pallidum,* and *T. pratense*. These findings indicate that the examined phenolic fractions may interfere with arachidonic acid cascade (Table 4).

**Table 4:** Effects of phenolic fractions of nine *Trifolium* species on malondialdehyde (MDA) generation in blood platelets stimulated with thrombin (0.4 U/ml) in vitro

*Trifolium* species [5 μg/ml]	MDA generation [nmol/ml of 2 × 10^8^ of platelets]
Control platelets	0.452 ± 0.043
*T. alexandrinum*	0.326 ± 0.023^**^
*T. hybridum*	0.374 ± 0.046^**^
*T. fragiferum*	0.398 ± 0.034
*T. incarnatum*	0.384 ± 0.026^*^
*T. pallidum*	0.405 ± 0.032^*^
*T. resupinatum* var. *resupinatum*	0.415 ± 0.033
*T. resupinatum* var. *majus*	0.410 ± 0.030
*T. scabrum*	0.379 ± 0.059
*T. pratense*	0.371 ± 0.023^**^

(*n* = 7; * *p* < 0.05, ** *p* < 0.01, as evaluated by the Dunnett and *t*-Student’s tests)

## Discussion

Our previous studies showed that *Trifolium*-derived plant extracts might display considerable antioxidant and free radical scavenging properties [20–23]. Besides the antioxidative action, the preliminary experiments on *T. pallidum* and *T. scabrum* (12.5–50 µg/ml) also suggested the possible inhibitory action of some clovers on platelet activation [32]. Therefore, in the present work, we extended our in vitro studies and analyzed the anti-platelet actions of phenolic fractions of nine clover species, with particular attention on the efficacy of lower concentrations of these preparations (1–5 µg/ml). The first step of our studies was to determine the anti-adhesive properties of the examined clovers with the use of different platelet agonists (ADP and thrombin) and fibrinogen as the adhesive surface, in an experimental system of isolated blood platelets. These variants allowed the comparative examination of the influence of plant extracts on the mechanisms of blood platelet adhesion. The adhesion of blood platelets to fibrinogen occurs via α_IIb_β_3_ integrin receptor. On resting platelets, α_IIb_β_3_ receptors are maintained in a low-affinity state, but during platelet activation, these receptors are rapidly transformed into a high-affinity conformation. Platelet response to ADP is mediated by its interactions with the G protein-linked P2 receptors (P2Y1 and P2Y12). Interaction of ADP with P2Y1 receptor, linked to G_q_, triggers the activation of phospholipase C (PLC), Ca^2+^ influx as well as mobilization of intracellular Ca^2+^. P2Y12, which is linked to Gi, leads to the inhibition of adenyl cyclase and decreases the cAMP level. The P2Y12 receptor is a target for several antagonists, used as anti-thrombotic/anti-platelet agents such as clopidogrel, prasugrel, ticagrelor, cangrelor, and elinogrel. Thrombin induces platelet activation due to protease-activated receptors (PARs): PAR-1 and PAR-4. The PARs are coupled to G_*q*_ protein, transmitting cellular signals mainly by stimulation of PLCβ [33–35].

The evaluation of anti-adhesive effects of *Trifolium* phenolic fractions indicated that *T. incarnatum*, *T. resupinatum* var. *majus,* and *T. scabrum* possess the strongest inhibitory effects on thrombin-induced adhesion to fibrinogen, contrary to the other preparations. Phenolic fractions of the remaining *Trifolium* species had little or no effects under those experimental conditions. Furthermore, phenolic fractions of the same *Trifolium* species as well as the preparation of *T. resupinatum* var. *resupinatum* were able to diminish platelet adhesion induced by ADP. Additionally, in experiments with ADP as an agonist, a statistically significant inhibition of platelet adhesion was also found for *T. pratense*.

Measurements of platelet adhesion in the presence of the *Trifolium* preparations were performed with the use of isolated human platelets, in a limited milieu. Therefore, the next analyses were conducted in PRP, in order to estimate clover effects under a more physiological environment. Both the platelet aggregometry and monitoring of platelet *α*-granules secretion process (PF-4 release) were performed using ADP or collagen as platelet agonists. The incorporation of collagen into experimental system enabled the evaluation of inhibitory effects of the examined phenolic fractions on platelet activation, occurring via specific platelet receptors for this protein. Blood platelets express several collagen receptors; the integrin receptor *α*2*β*1 is crucial for platelet adhesion to collagen surface, while GPVI and GPIb-IX-V are required for collagen-induced platelet activation [35, 36]. In this study, the ADP-induced platelet aggregation was inhibited by phenolic fraction of *T. resupinatum* var. *resupinatum* with the most efficacy; however, considerable inhibitory effects were also found for *T. alexandrinum*, *T. resupinatum* var. *majus,* and *T. scabrum* and, in some minor extent, for *T. fragiferum* and *T. pallidum*. The anti-platelet properties of *T. scabrum* were additionally confirmed by the determination of ADP-induced PF-4 secretion from platelet α-granules. Although some tendency seemed to be evident, the decline of PF-4 release in the presence of *T. resupinatum* var. *resupinatum, T. resupinatum* var. *majus,* and *T. pallidum* phenolics was statistically insignificant. On the other hand, the measurements of collagen-induced platelet aggregation demonstrated comparable inhibitory effects of the examined phenolic fractions, except the *T. alexandrinum* and *T. incarnatum*, which had no significant influence. Experiments with the collagen-induced PF-4 secretion from platelets revealed some inhibitory effects of *T. resupinatum* var. *resupinatum*, *T. resupinatum* var. *majus*, *T. scabrum,* and *T. pallidum*; however, the statistical significances of these differences were not found.

A general analysis of the obtained results indicates that phenolic fractions of *T. resupinatum* var. *majus*, *T. resupinatum* var. *resupinatum,* and *T. scabrum* displayed the strongest anti-platelet properties. Among all the tested preparations, the phenolic fraction of *T. scabrum* had the highest total phenolic content (80.93 mg/g of dry mass), including 72.76 mg/g of dry mass of isoflavones (which was the highest concentration of these substances as well). The preparations of *T. resupinatum* var. *resupinatum* and *T. resupinatum* var. *majus* were characterized by lower total phenolic content—17.32 and 22.54 mg/g of dry mass, respectively. However, the phenolic fraction of *T. resupinatum* var. *majus* contained (over two fold) higher concentration of isoflavones (11.17 mg/g of dry mass) when compared to the *T. resupinatum* var. *resupinatum* preparation (5.21 mg/g of dry mass). It is very likely that the presence of isoflavones significantly contributes to the anti-platelet action of these plant preparations. Although the role of isoflavones in modulation of blood platelet response has been little investigated, some evidence is available. Genistein and daidzein are able to inhibit the collagen-induced platelet aggregation in vitro [37]. Furthermore, Navarro-Núñez et al. [38] demonstrated that genistein might inhibit the platelet activation induced by thrombin. According to those authors, the anti-platelet action of genistein is a result of interfering with intracellular signaling and changes in calcium mobilization, but not an effect of interactions with thrombin receptors. It has been also shown that aglycones of soy isoflavones and equol are capable of binding to thromboxane A_2_ receptor in human platelets [39]. 8-Prenylnaringenin (an isoflavone derivative) possesses both anti-aggregatory and anti-adhesive effects on human platelets, independently on its interaction with estrogen receptors. It has been demonstrated that 8-prenylnaringenin is able to inhibit the phosphorylation of kinases such as Pyk2, Akt, and ERK1/2. The 8-prenylnaringenin-induced modulation of platelet response to agonists included inhibitory effect on ATP release from platelet dense granules as well as suppression of cytoplasmic Ca^2+^ mobilization [40].

On the other hand, the preparation from *T. alexandrinum*, containing much higher concentration of isoflavones (18.97 mg/g of dry mass) than phenolic fractions of *T. resupinatum* var. *majus* and *T. resupinatum* var. *resupinatum,* was less effective inhibitor of platelet activation of ADP- or thrombin-induced platelet adhesion, as well as in measurements of platelet aggregation and PF-4 secretion. These findings suggest an important role of other phenolic components, such as phenolic acids and different flavonoids that are present in the examined preparations from *Trifolium* species. According to the literature, numerous natural polyphenolic compounds display anti-platelet properties. For instance, caffeic acid may inhibit the collagen-induced aggregation of blood platelets. It is also able to reduce the generation of thromboxane A_2_, a physiological autocrine platelet agonist. Molecular mechanisms of this suppression involve the inhibition of Ca^2+^ mobilization, being a result of the cAMP-dependent phosphorylation of inositol-1,4,5-trisphosphate receptor [41]. The inhibitory effect on collagen-stimulated platelet functions was also found for quercetin, which may suppress activity of the Fyn kinase and phosphorylation of syk tyrosine kinase, and halt the PLC*γ*2 signaling [42]. Furthermore, three of the examined clover-derived preparations (of *T. alexandrinum*, *T. hybridum,* and *T. pallidum*) contained clovamides, an interesting group of caffeic acid derivatives. The presence of clovamide-type polyphenols was detected only in several plant genera, and clovers seem to be the most abundant source of these substances. Results from animal study indicated that clovamide (n-caffeoyldopamine) isolated from *Theobroma cacao* effectively reduced the expression of p-selectin and platelet-leukocyte interactions [43]. In our study, all of these three clovers displayed the inhibitory effect on thrombin-induced generation of MDA (Table 4), a marker of activation of arachidonic acid cascade, playing an important role in both the platelet activation and inflammatory processes. The decrease of MDA level was also found in platelets pre-incubated with phenolic fraction of *T. pratense*, which may confirm the anti-inflammatory action of this plant. The modulation of arachidonic acid pathway was previously reported in vitro by Lam et al. [25], who demonstrated the inhibitory effect of red clover isoflavones on COX-2 activity in murine and human monocyte/macrophage experimental systems.

The current knowledge of pharmacological activity of *Trifolium* plants is incomplete. Besides several reports containing studies on one or two clovers [44, 45], evaluation of wound healing properties of 13 clover species [46] and our previous examinations of antioxidant action of clovers, there is a lack of more extensive analysis covering at least several *Trifolium* species. In this in vitro study, for the first time, we evaluated and compared anti-platelet properties of nine *Trifolium* species, at concentration range, which may be partly relevant to the physiological conditions. Our experiments have demonstrated that the examined clovers display considerable inhibitory effects at the concentrations of 1–5 µg/ml. The plasma concentration of different plant-derived polyphenols is estimated at a range up to 3 µmol/l, or even about 4 µmol/l for some substances such as genistein, genistin, and gallic acid [47]. Studies on bioavailability of red clover isoflavones revealed that a single dose of dietary supplement, containing 38.8 mg of isoflavones yields the following plasma concentrations of these compounds: 0.35 μM for irilone, 0.39 μM for daidzein, and 0.06 μM for genistein [48].

## Conclusions

In conclusion, this work is the first comparative evaluation of anti-platelet properties of nine *Trifolium* species. Our studies revealed that the phenolic fractions of *T. scabrum*, *T. resupinatum* var. *resupinatum,* and *T. resupinatum* var. *majus* seem to be the most effective inhibitors of platelet activation; however, anti-platelet effects (in a minor extent) were also found for other of the examined species. The obtained results may be a base for further studies on clovers as a source of extracts with anti-platelet properties.
